# HindwingLib: A library of leaf beetle hindwings generated by Stable Diffusion and ControlNet

**DOI:** 10.1038/s41597-025-05010-y

**Published:** 2025-04-23

**Authors:** Yi Yang, WenJie Li, RuiZe Liu, ChengZhe Wu, Jing Ren, YiShi Shi, SiQin Ge

**Affiliations:** 1 Key Laboratory of Zoological Systematics and Evolution, Institute of Zoology, Chinese Academy of Sciences, 1 Beichen West Road, Chaoyang District, Beijing, 100101 China; 2 Department of Scientific Research, Beijing Planetarium, Xizhimenwai Street, Beijing, 100044 China; 3 University of Chinese Academy of Sciences, Beijing, 100049 China; 4 Center for Materials Science and Optoelectronics Engineering, University of Chinese Academy of Sciences, Beijing, 100049 China; 5 Aerospace Information Research Institute, Chinese Academy of Sciences, Beijing, 100094 China; 6 State Key Laboratory of Plant Genomics, Institute of Genetics and Developmental Biology, Chinese Academy of Sciences, Beijing, 100101 China

**Keywords:** Adaptive radiation, Optical imaging

## Abstract

The utilization of datasets from beetle hindwings is prevalent in research of morphology and evolution of beetles, serving as a valuable tool for comprehending the evolutionary processes and functional adaptations under specific environmental conditions. However, the collection of hindwing images of beetles poses several challenges, including limited sample availability, complex sample preparation procedures, and restricted public accessibility. Recently, a machine learning technique called Stable Diffusion has been developed to statistically generate diverse images using a pretrained model with prompts. In this study, we introduce an approach utilizing Stable diffusion and ControlNet for the generation of beetle hindwing images, along with the corresponding results obtained from its application to a diverse set of 200 leaf beetle hindwings. To demonstrate the fidelity of the synthetic hindwing images, we conducted a comprarative analysis of three key metrics: Structural Similarity Index (SSIM), Inception Score (IS), and Fréchet Inception Distance (FID), which are crucial for evaluating image fidelity. The results demonstrated a strong alignment between the actual data and the synthetic images, confirming their high fidelity. This novel library of leaf beetle hindwings not only offers morphological image for utilization in machine learning, but also showcases the extensive applicability of the proposed methodology.

## Background

As one of the most crucial functional organs in insects, wings play a pivotal role in insect flight and are considered a key feature contributing to the remarkable success of insect evolution^1,2^. Wing morphology serves as an indicator of the functional adaptation^3–5^ and evolutionary history of insects^6,7^. Beetles represent the most diverse group, with their specialized forewings and intricately folded hindwings^8^ being considered crucial morphological indicators that are both meaningful and indispensable for analyzing evolutionary patterns of wing morphology^9^.

The application of machine learning methodologies is widespread in entomological research, particularly in the domains of classification and detection. However, the effectiveness of these techniques is heavily reliant on the size of the training datasets available. Traditional machine learning model training is hindered by the scarcity of large-scale publicly accessible beetle hindwing landmark datasets. Moreover, compiling and annotating substantial datasets is an inherently labor-intensive and time-consuming endeavor^10^. Therefore, the introduction of data generation technology is highly desirable to address the issue of insufficient data on insect wings.

The utilization of GAN-based data generation methods has already been employed in insect research for the purpose of data augmentation, primarily focusing on classification and detection tasks. The DCGAN, WGAN, and VAE are widely recognized as the most prevalent generative methods for synthesize insect^11^. However, these approaches often face limitations due to the instability of adversarial training and their reliance on large-scale datasets, which are particularly challenging to acquire in specialized domains like beetle hindwing morphology^12^. In contrast, diffusion-based frameworks, such as Stable Diffusion combined with ControlNet, offer a more stable and data-efficient alternative. Recent advancements in diffusion models have demonstrated remarkable capabilities in generating biological images, such as generating high-resolution MRI from low-resolution counterparts^13^ and fully-annotated microscopy image datasets across various biological specimens^14^. Yet their application remains absent in specialized domains such as insect wing synthesis, where generative modeling could revolutionize morphological studies. By leveraging pre-trained text-to-image diffusion models and integrating spatial conditioning, our approach enables precise control over landmark-guided synthesis while requiring significantly fewer training samples.

The proposed approach leverages the Stable Diffusion model in conjunction with ControlNet to improve the efficiency of landmark dataset generation and directly addresses the limitations of existing approaches by facilitating the creation of extensive training datasets. The Stable Diffusion model, functioning as a pretrained large-scale model for image generation based on prompts^15^, exhibits strong creativity but lacks controllability. However, the incorporation of ControlNet, which has already been successfully employed in various forms of image generation, can address this limitation by enabling the generation of new hindwing images with adjustable landmarks^16^. The proposed approach was employed to augment the hindwing dataset, and we evaluate the performance of the augmented dataset. Encouragingly, the dataset exhibited promising fidelity. The main contributions of this study were as follows: 1) we proposed a novel approach for generating hindwing images with controllable landmark geometry, 2) we generated a augmented dataset with generated hindwing images which can improve the training of landmark detection networks. This approach would provide a novel perspective, contributing to an enhanced comprehension of the hindwings of beetles.

## Methods

### Hardware and Software Environment

The research utilized Python 3.8.0 (Python Software Foundation, Beaverton, OR, USA) and PyTorch 1.13.1 (Facebook, Inc., Menlo Park, CA, USA) for code implementation. Experiments were carried out on a graphics workstation running Ubuntu 18.04.1 LTS OS, equipped with an Intel(R) Xeon(R) Platinum 8160 CPU, 256 GB RAM, and a 24 GB NVIDIA GeForce RTX 3090 GPU. The corresponding versions of NVIDIA CUDA (NVIDIA Corporation, Santa Clara, CA, USA) and cuDNN (NVIDIA Corporation, Santa Clara, CA, USA) used were 11.6 and 8.3.2, respectively.

### Images and datasets preparation

**Fig. 1:**
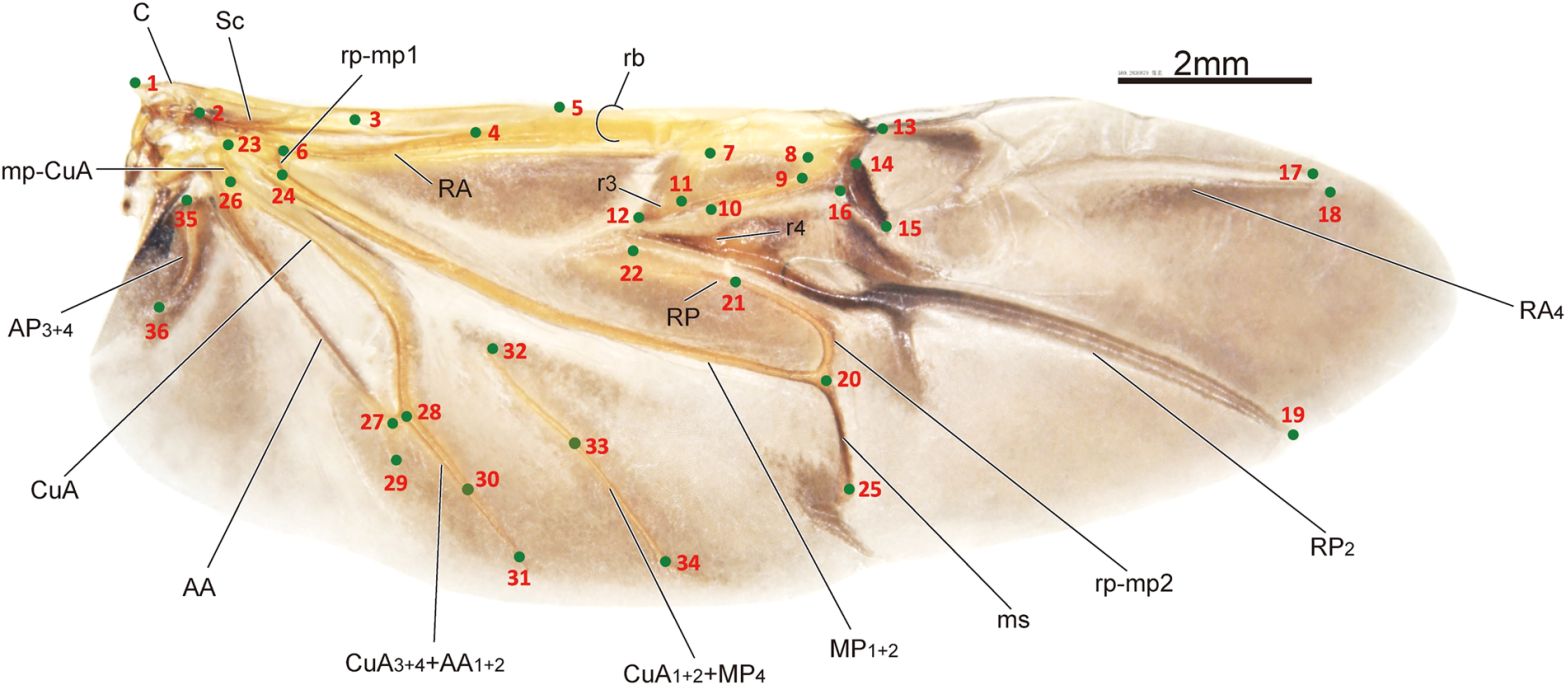
The distribution of the hindwing landmarks and the names of the hindwing veins for the leaf beetle (*Potaninia assamensis*)^30^.

The dataset comprises images of 256 leaf beetle hindwings, representing 16 subfamilies and 231 genera. The dataset consists of 36 identical landmarks Table 1, which were selected due to their biological significance and critical roles in the distribution of hindwing veins, encompassing intersections, bases, and terminations or origins of these veins Fig. 1. These landmarks serve as key reference points for understanding the morphological variation and evolutionary adaptations among leaf beetles^17^.

**Table 1:** Basic information about 36 landmarks on leaf beetle hindwings^30^.

Landmark index	Position description
1	Proximal anterior point of humeral plate (HP)
2	The crossing point of BSc and Sc
3	The point of Sc getting to bifurcate into ScA and ScP
4	The crossing point of ScP and RA
5	The crossing point of ScA and RA
6	The crossing point of rp-m1 and RA
7	Proximal anterior point of radial cell
8	Distal anterior point of radial cell
9	Distal posterior point of radial cell
10	Anterior point of r4 (or the crossing point of r4 and radial cell)
11	Proximal posterior point of radial cell
12	Proximal point of r3
13	Apical hinge
14	The anterior point of triangular area of radial cell’s distal side
15	The posterior point of triangular area of radial cell’s distal side
16	The proximal point of triangular area of radial cell’s distal side
17	The distal point of RA_4
18	The distal point of RA_1
19	The distal point of RP_2
20	The point of MP_1+2_ getting to bifurcate
21	The posterior point of r4, or the crossing point of r4 and rp-mp2
22	The proximal point of RP
23	Anterior point of mp-cua
24	The crossing point of rm-mp1and MP
25	The posterior of medial spur
26	Posterior point of mp-cua
27	The point of AA getting to bifurcate
28	The point of AA_1+2_ getting to fuse with CuA_3+4_
29	The posterior or distal point of AA_3+4_
30	The proximal point of cv
31	The posterior or distal point of AA_1+2_+CuA_3+4_
32	Anterior point of CuA1+2+MP4
33	The distal point of cv
34	Posterior point of CuA_1+2_+MP_4
35	The base point of AP_3+4_
36	The posterior point of AP_3+4_

The annotation structure adheres to COCO dataset guidelines^18^, comprising image information and landmark annotation details. Image data includes the index (“id”), path (“file_name”), width (“width”), and height (“height”). Landmark annotations feature several fields.

Images are in TIFF format, with dimensions of 4288 × 2848 pixels. The methodology for processing these images is detailed in previous research^17^. Specimen hindwings were obtained through careful dissection with a LEICA MZ 12.5 microscope, then photographed using a Nikon D500s camera attached to a Zeiss Stereo Discovery V12 stereoscope. The origin for landmark coordinates is the lower left corner of the image.

The landmark coordinate array (“Keypoints”) has a length of 3 *k*, where *k* is the number of landmarks (36 here). Each landmark includes an *x*, *y* coordinate, and a visibility flag (*v*), which is always redundant in this context as visibility is guaranteed. Landmarks are referenced from the top left, with coordinates adjusted based on image height. The bounding box (“Bbox”) identifies the hindwing’s position with the first two values for the upper-left point, followed by width and height^18^.

### Network Architecture

The utilization of text-to-image models provides unparalleled flexibility in directing the creative process through natural language. It can be effectively harnessed to generate images depicting specific unique concepts, modify their appearance, or compose them in new roles and novel scenes. And the process of generating novel outputs is guided by a control image derived from the single image provided by the user. A single image, when combined with fine-tuned control images, is sufficient to generate diverse samples and is of particular significance. In the following section, we provide an overview of the fundamental aspects involved in applying ControlNet to our leaf beetle hindwing image and landmark data generation.

#### Latent Diffusion Models

The LDM loss is then given by:

1 $${L}_{LDM}={E}_{z \sim \varepsilon (x),y,\varepsilon  \sim {\mathcal{N}}(0,1),t}\left[| | \varepsilon -{\varepsilon }_{\theta }({z}_{t},t,{c}_{\theta }(y))| {| }_{2}^{2}\right]$$

where *t* is the time step, *z*_*t*_ is the latent noised to time *t*, *ε* is the unscaled noise sample, and *ε*_*θ*_ is the denoising network. The objective here is to effectively remove the noise that has been added to a latent representation of an image. During the training process, *c*_*θ*_ and *ε*_*θ*_ are jointly optimized in order to minimize the LDM loss. During the inference process, a random noise tensor is sampled and iteratively denoised to generate a new image latent representation, *z*_0_. Finally, this latent representation is transformed into an image using the pre-trained decoder $x{\prime} =D({z}_{0})$.

#### Stable Diffusion model

**Fig. 2:**
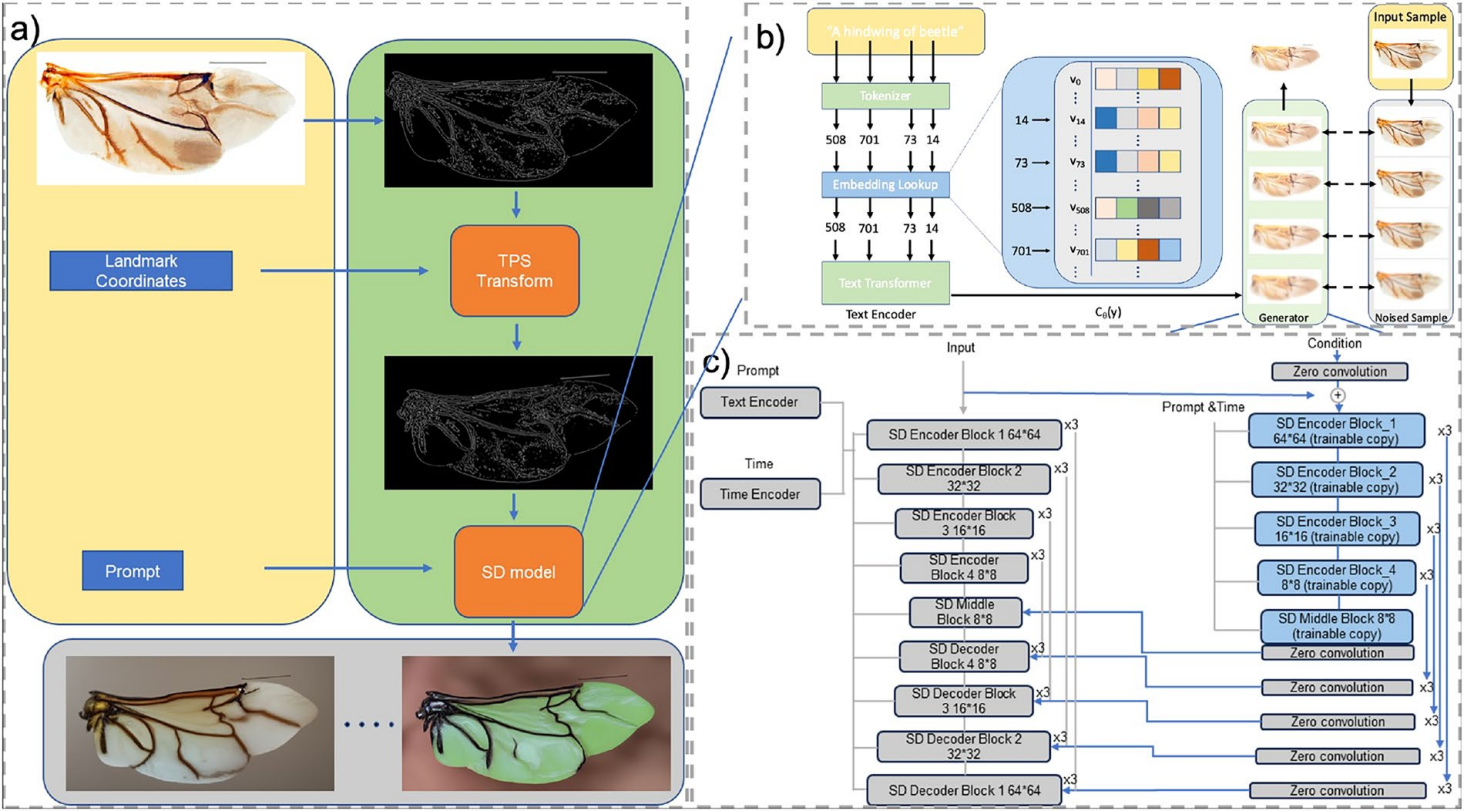
Procedure of the hingwing generation: **a**) Hindwing Generation Process: The process begins with applying the Canny edge detector to the reference hindwing image to produce an edge map. This edge map, along with landmark coordinates, serves as input for the Thin Plate Spline (TPS) transformation module, generating a new edge image with modified landmark positions. The altered edge image and a textual prompt are then provided to the Stable Diffusion (SD) model to produce a hindwing image reflecting the landmark adjustments. **b**) Stable Diffusion Architecture: A textual prompt is first tokenized into word or sub-word indices and converted into continuous embedding vectors. These embeddings are further transformed into a conditioning code *c*_*θ*_(*y*) that directs the generative model during image synthesis. **c**) ControlNet Integration with Stable Diffusion: The Stable Diffusion model’s U-Net architecture is depicted with gray blocks, while ControlNet modules are shown in blue. ControlNet introduces additional layers that incorporate conditional inputs, enabling controlled generation of images while maintaining structural consistency. The repeated module structure within ControlNet aligns with Stable Diffusion’s layers, facilitating the progressive denoising process and ensuring that wing vein contours remain intact throughout image generation.

The Stable Diffusion model, a large-scale implementation of latent diffusion^15^, is engineered for text-to-image generation tasks. It encodes textual prompts into latent embedding vectors using a pretrained CLIP model^19^, which has been trained on a diverse dataset of 512 × 512 images from the LAION-5B database. The architecture of Stable Diffusion, illustrated in Fig. 2b, employs a U-Net comprising an encoder, a middle block, and a skip-connected decoder. The model processes text prompts by converting them into tokens, embedding these tokens into continuous vectors, and transforming them into a conditioning code *c*_*θ*_(*y*) that guides the generative process. The U-Net architecture consists of 12 encoder blocks and 12 decoder blocks, with intermediate Vision Transformer (ViT) layers facilitating cross-attention mechanisms. During inference, a noise tensor is iteratively denoised through these layers to generate a coherent latent representation, which is then decoded into an image by the pretrained VAE decoder.

#### ControlNet

To enhance the controllability of the generated images, especially concerning the specific geometric distribution of beetle hindwing venation, we integrate ControlNet into the Stable Diffusion framework. As depicted in Fig. 2c, ControlNet modifies the Stable Diffusion model by introducing additional network layers that process conditional inputs, such as edge maps extracted from hindwing images. These conditional layers are designed with a hierarchical structure that mirrors the U-Net architecture, allowing for stepwise denoising while maintaining the integrity of the wing vein contours. Furthermore, the ControlNet architecture features repeated modules corresponding to the layers within the Stable Diffusion model’s U-Net. This repetitive, hierarchical structure is crucial for the stepwise denoising process characteristic of latent diffusion models. By aligning ControlNet’s layers with those of Stable Diffusion, ControlNet can effectively guide each denoising stage, progressively transforming noise into a clear, detailed image. This integration ensures that the geometric contours of the hindwing venation remain unchanged, thereby maintaining the structural fidelity of the beetle hindwings while producing high-quality images.

#### Generation of augmented landmark data set using Stable Diffusion and ControlNet

As illustrated in Fig. 2a, the Canny edge detection method is applied to a given hindwing image to generate its corresponding edge map using ControlNet. To create variability, random offsets are introduced to 36 designated landmarks on the hindwing, resulting in a new set of landmark coordinates. These original and offset coordinates are used as reference points in a Thin Plate Spline (TPS) transformation, which locally deforms the edge map to reflect the altered landmark positions. The deformed edge map serves as the conditional input for ControlNet, accompanied by the text prompt “a hindwing extracted from body.” The Stable Diffusion model then utilizes these inputs to generate a synthesized hindwing image that incorporates the adjusted landmark positions.

**Fig. 3:**
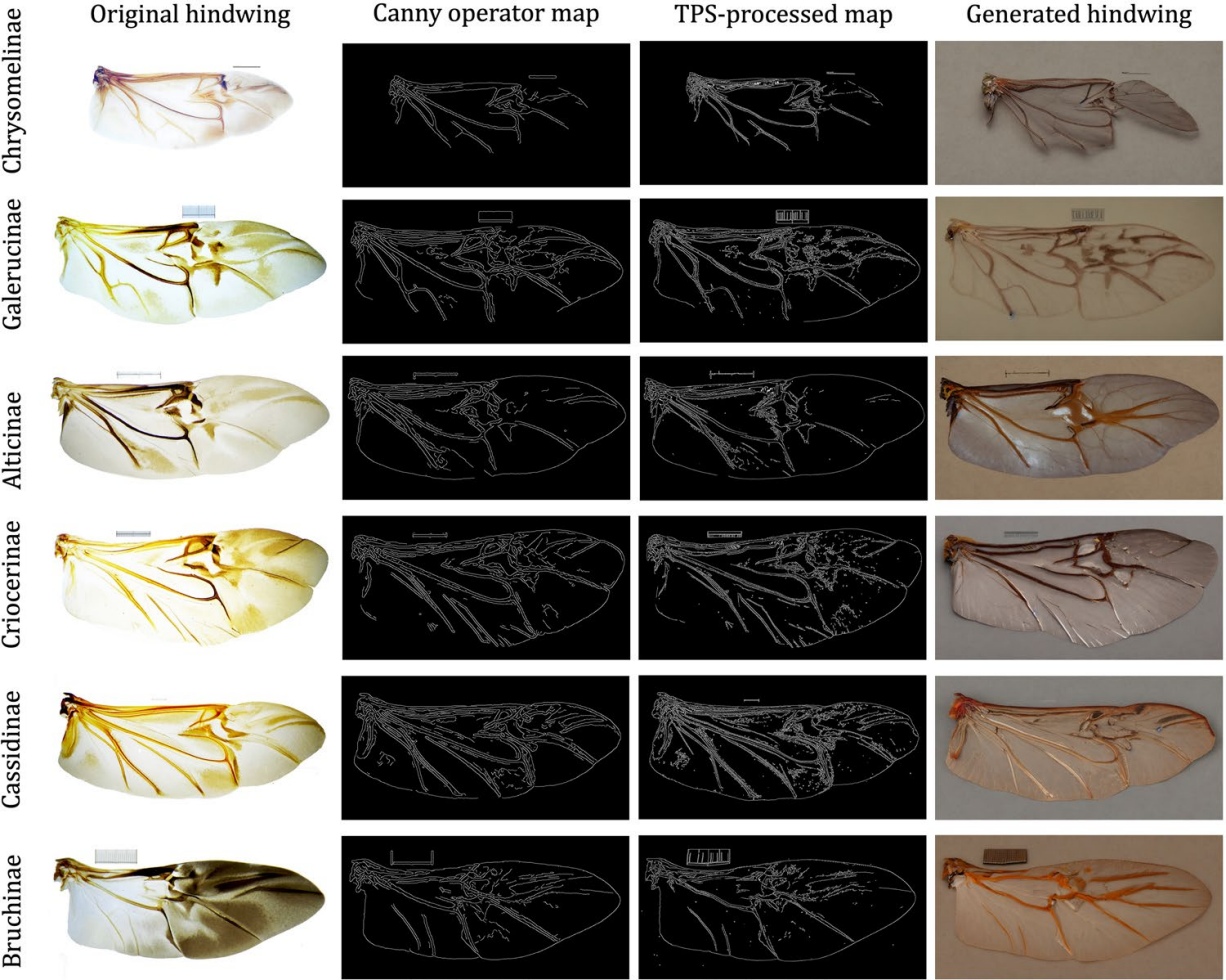
A selection of representative leaf beetle hindwing images are shown, where each row depicts a species, as well as each key stage of data processing required followed by the final generated images. Starting with image preparation and conversion, proceeding through operator map application, landmark extraction and adjustment, TPS-based deformation, and culminating in stable diffusion-driven image generation, each step illustrates progressive transformation in plant beetle hindwing processing.

The creation of a comprehensive database follows these steps Fig. 3:

1. Image Resizing and Conversion: Use an image scaling script to resize the tif images to 512  × 1024 pixels and convert them to png format.
2. Operator map generation: A group of operators are applied to generate a map on the hindwing image. This map highlights key features and landmarks on the wing.
3. Landmark Extraction and Adjustment: Extract the coordinates of landmarks along the hindwing veins. Adjust these coordinates by adding an offset. The offset is drawn from a Gaussian distribution with a mean of 0 and a standard deviation of 10 pixels.
4. Local Deformation using Thin Plate Spline (TPS): Apply TPS to locally deform the operator map. The original and offset coordinates serve as the reference points before and after transformation.
5. ControlNet and Image Generation: Use the deformed detected_map as the control condition for ControlNet. Generate a new hindwing image with the prompt “A hindwing extracted from body” for stable diffusion.

The procedures can be replicated by cloning the BeetleHindwing repository and executing main.py.

## Data Record

The full dataset can be accessed on Zenodo (10.5281/zenodo.10889131)^20^. The dataset is compressed into a single zip file, which includes two sub-folders of images and annotations. The images folder includes sub-folders representing four operators. Each operator folder comprises of eight sub-folders for the subfamilies of leaf beetles, with the sub-folder names corresponding to the subfamily names. Each of these subfolders contains media files (.png) for illustrating the generated images of the hindwings of the leaf beetles. They also include intermediate operator images and TPS (Thin Plate Spline) interpolated images, allowing users to generate new images.

**Fig. 4:**
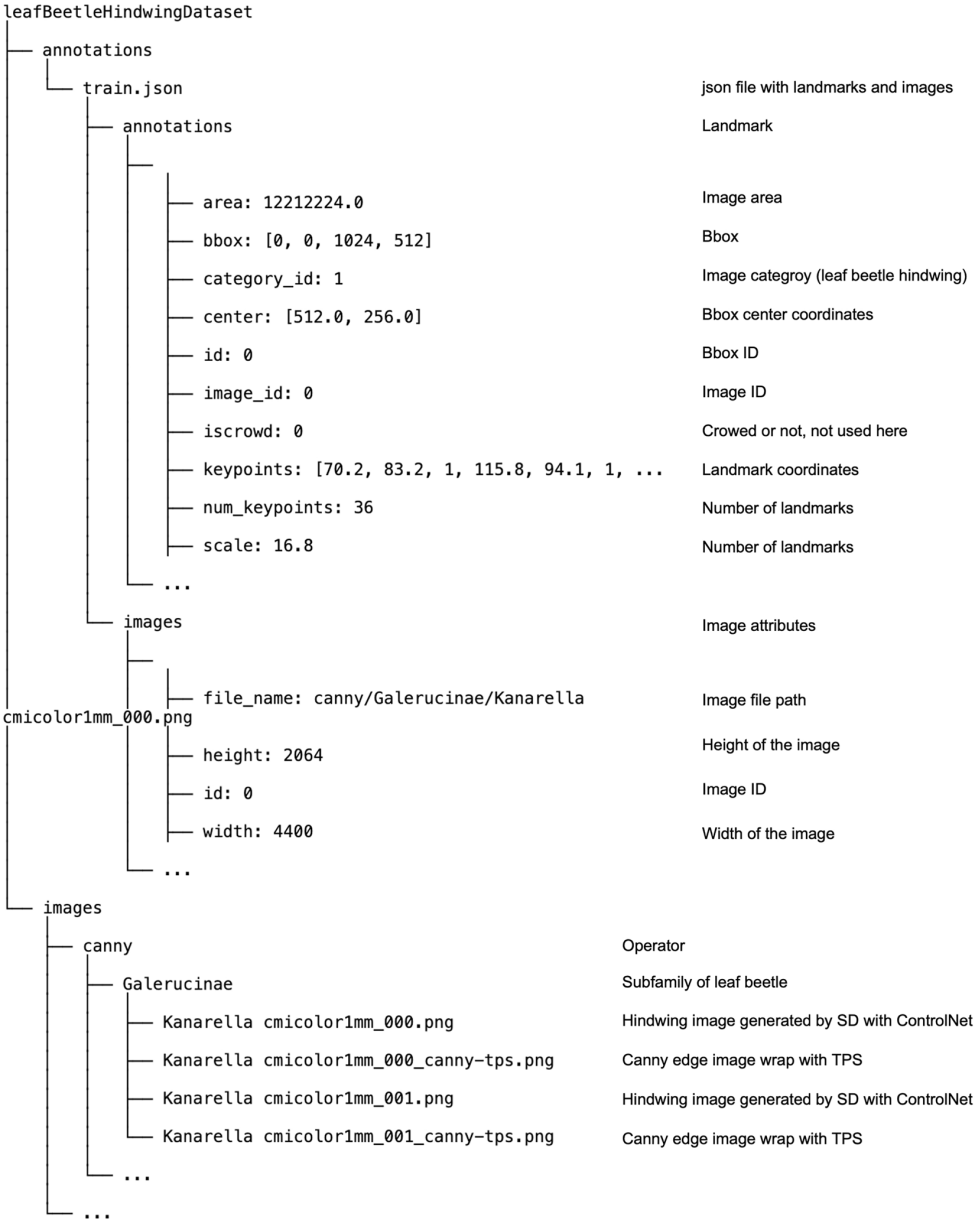
The directory tree of the dataset and a brief description of each file.

In addition to the folder images, the folder annotations contains an annotation file, train.json. This json file provides annotation information about the size of each leaf beetle hindwing image and the coordinates of landmarks on the corresponding hindwing. The complete directory tree and a brief description of each file are shown in Fig. 4.

## Technical Validation

### Comparison of the Performance of 4 ControlNet Pre-trained Models

**Fig. 5:**
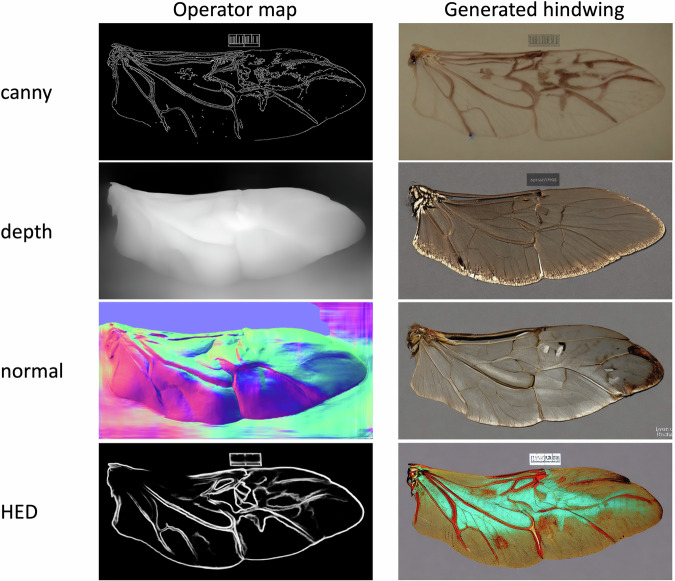
The generation effect of the hindwings of the beetle corresponding to four operator-controlled charts in ControlNet + SD is presented.

Based on Potaninia assamensis (Chrysomelinae), we employed four operators, namely canny, depth, normal, HED, to generate control images (as shown in Fig. 5) as input of the ControlNet + SD. All prompts are set to “a hindwing extracted from body”, resulting in the generated images shown as in Fig. 5. It is evident that the images controlled by canny and HED closely resemble bettle hindwings, while those controlled by depth and normal exhibit excessive collateral branches.

### Comparison of the performance of hindwings in different subfamilies of leaf beetle

The prompt should be set as “a hindwing extracted from body” using the operator being canny + control_v11p_sd15_canny. The generated image size was set to 1024 × 512. The “Control mode” was set to “Balanced”. The results of generating the hindwings of six subfamilies of leaf beetles are displayed in Fig. 3 using the canny operator with ControlNet and SD. The generated images of Galerucinae, Alticinae, Criocerinae, Cassidinae, and Bruchinae exhibit a high degree of realism and completeness, and only the image of Chrysomelinae appears to be lacking in completeness.

### Similarity between the generated and real hindwing images

The performance evaluation of ControlNet + Stable-Diffusion in hindwing image generation involves the utilization of Structural SIMilarity (SSIM), Inception score (IS), Fréchet Inception Distance (FID)^21^, and Learned Perceptual Image Patch Similarity (LPIPS)^22^ to quantify the resemblance between the similarity of generated images to real ones.

The IS metric is employed to assess the quality of the generated hindwing images. This metric serves as an effective evaluation tool, exhibiting a strong correlation with human judgment. It is defined as follows:

2 $$\exp ({{\mathbb{E}}}_{{\boldsymbol{x}}}\,{\rm{KL}}\,(p(y| {\boldsymbol{x}})| | p(y)))$$

where images that contain meaningful objects should have a conditional label distribution *p*(*y*∣***x***) with low entropy, and the model generated varied images should have a marginal distribution ∫ *p*(*y*∣***x*** = *G*(*z*))*dz* with high entropy. The metric is exponentiated to facilitate easier comparison. The pretrained Inception model [http://download.tensorflow.org/models/image/imagenet/inception-2015-12-05.tgz]^23^ is employed for each generated image to obtain the conditional label distribution *p*(*y∣****x***) as described by Salimans *et al*.^24^.

The FID metric exhibits greater consistency with the noise level compared to the IS. We call the Fréchet distance *d*(.,.) between the Gaussian with mean (*m*, *C*) obtained from *p*(.) and the Gaussian with mean (*m*_*w*_, *C*_*w*_) obtained from *p*(.)_*w*_ the “Fréchet Inception Distance” (FID), which is given by^25^:

3 $${d}^{2}((m,C),({m}_{w},{C}_{w}))=| | m-{m}_{w}| {| }_{2}^{2}+\,{\rm{Tr}}\,(C+{C}_{w}-2{(C{C}_{w})}^{1/2})$$

The FID computation involved propagating all images from the training dataset through the pretrained Inception-v3 model, following the calculation of the Inception Score^24^. The last pooling layer is utilized as the coding layer, following with the calculation of the mean *m*_*w*_ and the covariance matrix *C*_*w*_.

We employ LPIPS with 5 conv layers from the VGG network, which has become the established standard for image generation tasks^26–28^. Specifically, the conv1-conv5 layers are as described in^29^.

The LPIPS is calculated as the distance between reference and distorted patches *x*, *x*_0_ using a network. The feature stack is extracted from *L* layers and unit-normalized in the channel dimension, which are designated as ${\widehat{y}}^{l},{\widehat{y}}_{0}^{l}\in {{\mathbb{R}}}^{{H}_{l}\times }$W_l_ × C_l_}\) for layer *l*. The activations are scaled channel-wise by a vector ${w}^{l}\in {{\mathbb{R}}}^{{C}_{l}}$ and the *ℓ*_2_ distance is computed. The final step involves spatial average and channel-wise summation.

4 $$d(x,{x}_{0})=\sum _{l}\frac{1}{{H}_{l}{W}_{l}}\sum _{h,w}| | {w}_{l}\cdot ({\widehat{y}}_{hw}^{l}-{\widehat{y}}_{{0}_{hw}^{l}})| {| }_{2}^{2}$$

**Fig. 6:**
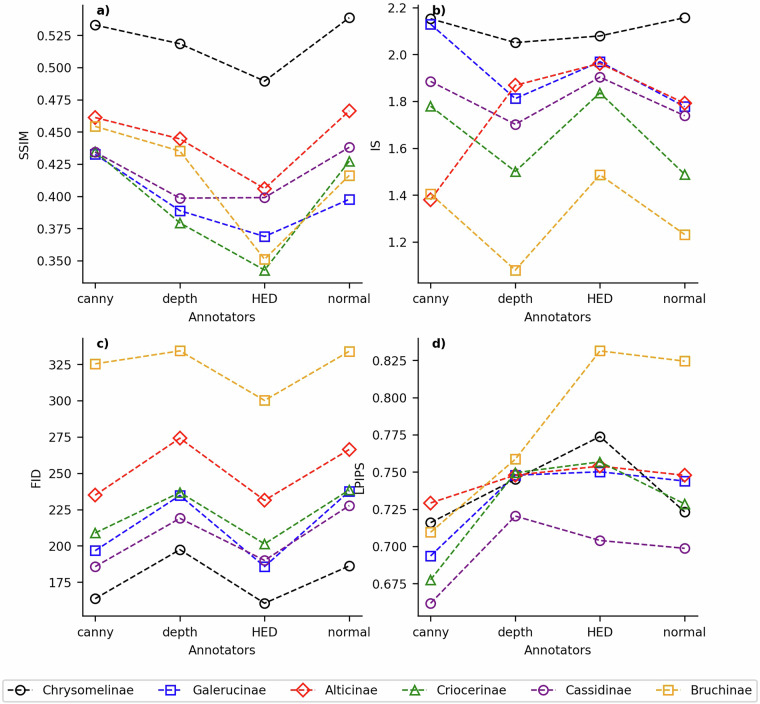
Four similarity metrics of four operators on six subfamilies.

The generated image dataset is evaluated using SSIM, IS, FID, and LPIPS metrics in Fig. 6a–d to quantify the similarity between the generated and original image datasets. The figures demonstrate that the Chrysomelinae subfamily exhibits the highest level of IS, the lowest FID, and relatively low LPIPS scores, indicating that images generated from this subfamily possess superior realism. On the contrary, the Bruchinae subfamily exhibits the lowest IS, highest FID, and relatively high LPIPS scores, suggesting that the generated images from this particular subfamily possess diminished realism. The lower realism scores observed for the Bruchinae subfamily can be primarily attributed to the limited number and quality of the input samples available. The photographs of Bruchinae specimens are less clear and suffer from uneven lighting conditions, which may introduce greater deviations during intermediate processing stages such as the extraction of vein patterns. Since these vein patterns serve as a crucial input to the ControlNet model, which dictates the contours of the generated images, any deviation can lead to distorted outputs. This distortion is the key reason behind the lower realism scores for the Bruchinae. To ensure the generation of high-quality images, it is essential to acquire high-quality input sample images.

The proposed approach can be effectively extended to other types of insect wings, provided that appropriate image datasets are collected. This approach holds the potential to significantly enhance our understanding and documentation of insect morphology across a wide array of species. By leveraging ControlNet’s capabilities to generate realistic and detailed imagery, researchers can explore morphological variations and adaptations in other insect groups, ultimately contributing to the fields of taxonomy, evolutionary biology, and ecological studies. This adaptability underscores the broad applicability of our method and supports its use as a valuable tool for entomologists and other researchers working with limited or challenging datasets.

## Data Availability

Code is available on Github https://github.com/mgcyung/BeetleHindwing.
